# The transcaval approach may be the new route vascular surgeons need in their arsenal

**DOI:** 10.1016/j.jvscit.2026.102143

**Published:** 2026-01-09

**Authors:** Mathew J. Kahn, Mina L. Boutrous

**Affiliations:** Division of Vascular and Endovascular Surgery, University of Connecticut, Farmington, CT

Cambiaghi et al^1^ present on the adaptation of transcaval access for delivery of a branched endograft through a large-bore delivery sheath. The authors believe that transcaval access can become a more mainstream method of dealing with difficult iliofemoral anatomy in the vascular community.^1^

Conventional methods for dealing with inadequate access vessel size for endovascular procedures depend on the nature and extent of the stenosis, occlusion, or diminutive segment. Techniques such as balloon angioplasty, stenting, lithotripsy, serial dilation, and endarterectomy are effective for isolated insults.^2^ The use of open iliac or direct aortic conduits via retroperitoneal approach, is well described in the literature and has been documented to be necessary in approximately 15% to 17% of thoracic endovascular aneurysm repair candidates. Open conduits have a reported technical success of ≤95% and can be ligated or converted to iliofemoral bypasses once the endovascular graft is deployed. However, the use of open conduits are linked to higher rates of perioperative complications, longer hospital stays, and higher 30-day mortality rates than an endovascular-only procedure, making this approach less appealing in patients deemed at high risk for open surgery.^2^, ^3^, ^4^ An entirely endovascular solution to inadequate iliofemoral access is the pave and crack endoconduit technique. Iliac arteries are lined with covered stents and balloon angioplasty dilation is used to create a controlled rupture. In one single-center trial, this technique has been shown to be effective against even TransAtlantic Inter-Society Consensus D lesions and had 100% success rate in delivering large bore sheaths.^5^ One significant limitation of this method includes the frequent need to cover hypogastric arteries, leading to increased risk of spinal cord or pelvic ischemia.

Although seldom used for aortic interventions, there is extensive literature covering transcaval aortic access for transcatheter aortic valve implantation in those with poor iliofemoral access vessels. Compared with transapical, transaxillary, and transcarotid approaches, the transcaval aortic access is a fully endovascular method with lower perioperative mortality, comparable stroke/transient ischemic attack rates, and a technical success of 97.5%.^6^ Anatomical requirements of the infrarenal aorta include a 10- to 15-mm calcium-free segment adjacent to the inferior vena cava, <30° of leftward angulation, and the absence of a pedunculated thrombus. Closure of the access site is achieved through the use of nitinol cardiac occlusion, which often creates a controlled aortocaval fistula, rather than completely closing the defect.^7^

The case report by Cambiaghi et al offers a relatively novel and promising solution for delivery of a thoracoabdominal endoprosthesis in patients with prohibitive iliofemoral anatomy. There are several considerations that should be at the forefront of future studies focused on adapting transcaval aortic access for use with a thoracoabdominal branch endoprosthesis (TAMBE). Delivery of thoracoabdominal component of the TAMBE requires a much larger sheath than is typically used. The window for aortocaval crossing in a TAMBE procedure is also highly limited to a small segment of aorta just proximal to the iliac bifurcation to facilitate complete deployment of thoracoabdominal component, which often carries a heavier atherosclerotic burden. It is also imperative to note that, given the rarity of this type of access, very few trainees are exposed to it, further restricting its familiarity in real-world practice. This case report serves to facilitate discussion and further clarify the direction future studies should focus on. With time, transcaval aortic access has the potential to become a valuable addition to the vascular surgeon's toolbox when treating aortic pathologies in patients with challenging access vessels.

*The opinions or views expressed in this commentary are those of the authors and do not necessarily reflect the opinions or recommendations of the Journal of Vascular Surgery Cases and Innovative Techniques or the Society for Vascular Surgery*.

## Funding

None.

## Disclosures

None.
